# Oxidative stress and age-related olfactory memory impairment in the honeybee *Apis mellifera*

**DOI:** 10.3389/fgene.2014.00060

**Published:** 2014-03-25

**Authors:** Tahira Farooqui

**Affiliations:** Department of Entomology, The Ohio State UniversityColumbus, OH, USA

**Keywords:** oxidative stress, memory formation, olfactory dysfunction, Parkinson disease, Alzheimer's disease, *Apis mellifera*

The honeybee (*Apis mellifera*) olfactory system is well adapted to detect and discriminate a diverse array of odors. *Apis mellifera* is one of the most important insect species to study olfactory learning and memory because it can be conditioned to respond with feeding movements of the mouthparts (proboscis) to a variety of floral odors by using Proboscis Extension Reflex (PER) conditioning. Honeybee workers switch from in-hive (nursing) to outside-hive (foraging) tasks depending on their age, colony demand, and outside conditions. Workers use their sophisticated cognitive abilities in both foraging, and task performance within the colony (Menzel and Giurfa, 2001; Frasnelli et al., 2014). The honeybee olfactory system possesses olfactory sensory neurons inside the cuticle-covered sensillae along the antennae, which are equivalent to olfactory epithelia within the nasal cavity in vertebrates. From antenna the information is carried via axons of olfactory sensory neurons directly to the antennal lobe (AL) that is equivalent to the olfactory bulb (OB) in vertebrates. This information is processed in the AL then relayed by projection neurons to the mushroom bodies (MB), which contribute to memory consolidation associated with long-term potentiation and synaptic organization (Hourcade et al., 2010). The exhibiting synaptic plasticity in the MB is similar to that of mammalian hippocampal synaptic plasticity (Bliss and Collingridge, 1993).

The neuronal synaptic plasticity can be regulated by important signaling molecules called as “biogenic amines” that modulate synaptic morphology, number of synapses, and receptors, influencing animal behaviors including complex behaviors such as learning and memory formation in both vertebrates and invertebrates. Prominent examples of biogenic amines include epinephrine, norepinephrine, dopamine, serotonin, octopamine, and tyramine. Norepinephrine and epinephrine are preferentially synthesized by vertebrates; whereas octopamine and tyramine are preferentially synthesized by invertebrates. Biogenic amines exert their activity by interacting with specific G-protein coupled receptors, causing changes in the levels of intracellular second messengers (Scheiner et al., 2006). In vertebrates, both the OB and cortex receive heavy inputs from cholinergic, noradrenergic, and serotonergic modulatory systems, exerting profound effects on both odor processing and odor memory by acting on both inhibitory local interneurons γ-amino butyric acid (GABA) and output neurons in both regions (Fletcher and Chen, 2010). The primary sensory afferents from the olfactory neuroepithelium to OB can be modulated by a presynaptic inhibition-mediated by GABA and dopamine released from bulbar interneurons. Increased levels of octopamine in the AL mediate an important role in a reinforcement pathway involving olfactory learning and memory in *Apis mellifera* (Farooqui et al., 2003). Both dopamine and serotonin exert dual roles in appetitive and aversive olfactory memory (Sitaraman et al., 2012; Waddell, 2013); and dopamine and octopamine mediate differential modulation of nicotine-induced calcium in MB kenyon cells (Leyton et al., 2014) in *Drosophila melanogaster*. These findings suggest that biogenic amines play neuromodulatory roles in memory formation in insects like mammals.

**Figure 1:**
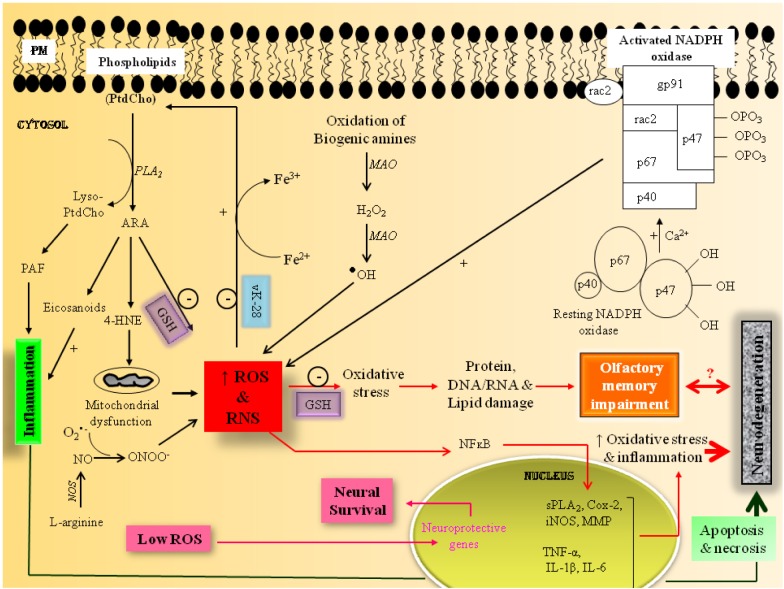
**Hypothetical scheme showing the potential molecular mechanisms involved with oxidative stress-mediated impairment of olfactory memory**.

Oxidative stress occurs due to a disturbance in the cellular pro-oxidant/antioxidant ratio. It is characterized by increase in reactive oxygen species (ROS) and reactive nitrogen species (RNS), as well as depletion of antioxidant levels. ROS include superoxide anions, hydroxyl and peroxyl radicals, and hydrogen peroxide (H_2_O_2_), which are generated as by-product of oxidative metabolism (Massaad and Klann, 2011). The major sources of ROS in brain include mitochondrial respiratory chain, uncontrolled arachidonic acid (ARA) cascade, and activation of NADPH oxidase (Figure 1). Another mitochondrial source of ROS is the enzyme family of monoamine oxidases (MAO) that catalyze the oxidation of biogenic amines, generating free radicals. Like ROS, RNS are formed during amino acid metabolism with the generation of nitric oxide (NO), which reacts with superoxide radical (O^•−^_2_) to form peroxynitrite (ONOO^−^) that interacts with lipids, DNA, and proteins, disrupting cell signaling to overwhelming oxidative and nitrosative injury (Pacher et al., 2007).

In neural cells, ROS modulate gene expression through phosphorylation, activation and oxidation of transcription factors, adhesion molecules and chemotactic factors, antioxidant enzymes, and vasoactive substances such as Nuclear factor erythroid 2-related factor 2 (Nrf2), activator protein 1 (AP1), nuclear factor-kappaB (NF-kB), FOXO transcription factors (FOXO), hypoxia-inducible factor-1alpha (HIF-1α), p53, and heat shock proteins (Hsps) (Massaad and Klann, 2011). Many of these transcription factors contain redox-sensitive cysteine residues in their DNA binding sites. Oxidative modifications of these residues affect DNA binding and subsequently regulate gene transcription of redox sensitive genes. Low levels of ROS induce Nrf2, a potent transcription factor, which modulates genes for NADPH quinone oxidoreductase (NQO1), glutathione S-transferase, heme oxygenase-1 (HO-1), ferritin, and γ-glutamylcysteine synthetase. Similarly, moderate increase in ROS results in activation of transcription factor AP-1, which modulates immediate early genes (c-fos and c-jun). The activation of Nrf2 and AP1 modulates transcription of neuroprotective genes, contributing to neural cell survival. In contrast, high levels of ROS activate transcription factor, NF-κ B, which modulates the transcription of proinflammatory cytokines (TNF-α, IL-1β, IL-6) and enzymes (cPLA_2_; COX-2, LOX, iNOS, MMP). High levels of ROS accumulation results in opening of membrane permeability transition pore, and release of cytochrome c from the mitochondria, leading to apoptotic cell death (Figure 1). Excessive ROS decrease cognitive performance in mammals (Massaad and Klann, 2011) as well as in honeybees (Farooqui, 2008; Williams et al., 2008). Excessive ROS and RNS production is associated with mitochondrial dysfunction-mediated neuronal damage, which involves deficits in olfactory memory in neurodegenerative diseases such as Parkinson's disease (PD) and Alzheimer's disease (AD) (Haehner et al., 2007; Bahar-Fuchs et al., 2011). The olfactory nervous systems of insects and mammals exhibit many anatomical and physiological similarities. Therefore, purpose of present opinion is to validate whether honeybee can serve as an excellent tool for studying candidate genes responsible for ROS-mediated olfactory dysfunction in aging and neurodegenerative diseases.

Biogenic amines like octopamine or dopamine modify neural function at multiple levels, modifying insect behavior. Octopamine or dopamine can substitute for the appetitive and aversive reinforcements, respectively. The disruption of octopamine receptor (AmOA1) in the AL of the honeybee brain inhibits olfactory acquisition and recall without altering odor discrimination, suggesting that octopamine mediates consolidation of a component of olfactory memory at this early processing stage (Farooqui et al., 2003). However, in the MB disruption of AmOA1 does not affect acquisition but significantly inhibits recall, implicating that octopamine is not involved in early stages of immediate memory encoding in the MB (Farooqui et al., unpublished). Age-dependent changes in brain biogenic amine levels are associated with morphological development and behavioral plasticity in honeybees (Harris and Woodring, 1992). A marked deficit in olfactory learning and memory in honeybees can be achieved by the flight activity, environmental and physiological stress (Morimoto et al., 2011), aging (Farooqui, 2007) and presence of excessive iron in the brain (Farooqui, 2008), implicating a link between ROS-mediated oxidative stress and olfactory dysfunction. Similarly, increase in oxidative stress in the olfactory epithelium of AD patients is accompanied by olfactory impairment (Getchell et al., 2003; Perry et al., 2003). The abnormal levels of biogenic amines and disruption in signal transduction also seem to be linked with olfactory memory defecits in PD and AD (Marien et al., 2004). Thus, ROS-mediated mechanisms underlying olfactory dysfunction in mammals and honeybees seem to be analogous.

A variety of model systems are available for aging and neurodegenerative diseases. Fundamental processes in models can be shared with humans; however, models can never capture the full complexity of the human condition. Genetic models fail to phenocopy the human diseases in that they generally lack a behavioral phenotype and/or the characteristic pathological feature of human disease. Moreover, there is a distinct inability of either approach to model sporadic, late-onset neurodegenerative disease, which accounts for over 90% of human cases. In spite of limitations, models have been used to study the molecular mechanism of brain diseases. *Drosophila melanogaster* has provided significant insights into the mechanisms of learning and memory using genetic approaches combined with molecular, anatomical, and behavioral tools (Davis, 2004). However, *Drosophila* is not capable of producing higher order cognitive behaviors like humans. In contrast, *Apis mellifera* has been shown to be a great model in cognitive neuroscience because of its sophisticated cognitive abilities (Menzel and Giurfa, 2001; Frasnelli et al., 2014). The life span of honeybee workers can range from 6 weeks to more than 6 months depending on season, and temporal caste (Omholt and Amdam, 2004). All workers can have similar genotypes due to genetic manipulation; therefore their life span is mainly regulated by environmental factors. *Apis mellifera* genome is evolved more slowly and more similar to vertebrates for circadian rhythm, RNA interference, DNA methylation, and learning and memory genes than other insects. It has fewer genes for innate immunity, detoxification enzymes, cuticle-forming proteins, and gustatory receptors, but more odorant receptor genes compared to fruit fly and mosquito, and novel genes for nectar and pollen utilization (Weinstock et al., 2006). *Apis mellifera* brain contains only one million neurons (five orders of magnitude less than the human brain but four times greater than *Drosophila*). Oxidative stress can be successfully induced in honeybee worker brain. It is also amenabile to molecular, genetic, pharmacological, and social manipulations. These characteristics favor *Apis mellifera* worker for serving as an excellent tool for testing the role of candidate genes for ROS-mediated olfactory dysfunction. This may lead to better understanding of molecular mechanisms involved with olfactory dysfunction in human with aging and neurodegenerative diseases.
